# The Presence of Visual Neglect after Thrombolytic Treatment in Patients with Right Hemisphere Stroke

**DOI:** 10.1100/2012/434120

**Published:** 2012-02-02

**Authors:** J. E. Kettunen, M. Nurmi, A.-M. Koivisto, P. Dastidar, M. Jehkonen

**Affiliations:** ^1^Deparment of Neurology and Rehabilitation, Tampere University Hospital, P. O. Box 2000, Tampere FIN-33521, Finland; ^2^School of Social Sciences and Humanities, Psychology, University of Tampere, Tampere FIN-33014, Finland; ^3^Tampere School of Public Health, University of Tampere, Tampere FIN-33014, Finland; ^4^Regional Medical Imaging Center and Tampere Medical School, Tampere University Hospital, P. O. Box 2000, Tampere FIN-33521, Finland

## Abstract

Visual neglect (VN) is a common consequence of right hemisphere (RH) stroke. The aims of this study were to explore the presence of VN after RH stroke in the patients with (T+) or without (T−) thrombolytic treatment, and to determine whether thrombolysis is a predictor of VN. The study group consisted of 77 RH infarct patients. VN was evaluated with six conventional subtests of the Behavioural Inattention Test (BIT). Stroke severity was assessed using the National Institute of Health Stroke Scale (NIHSS). In the neuropsychological examination, 22% of all RH stroke patients had VN. VN was present in 15% of the patients in the T+ group and in 28% of the patients in the T− group, but the difference was not statistically significant. Despite that, patients in the T− group had a higher risk of VN than patients in the T+ group. Our results suggest that thrombolysis independently predicted absence of VN.

## 1. Introduction

Previous studies have confirmed that visual neglect (VN) is a common consequence of right hemisphere (RH) stroke [1–5]. The prevalence of VN has been studied extensively, and the results of this work are summarized in a review by Bowen et al. [1]. According to Bowen et al. [1], the median reported prevalence of neglect in RH patients is 43%. The presence of neglect implies a poor prognosis in terms of discharge time, length of hospital stay, and functional recovery [6–8].

Previous studies [9, 10] have shown that patients who receive thrombolytic treatment within the first three hours of ischemic stroke can expect a favourable or good three-month clinical outcome. The benefit of thrombolytic treatment is thought to be due to vessel recanalization resulting in restitution of blood flow to ischemic regions of brain [11, 12] which leads to neurological improvement, smaller infarct size, and better clinical outcome [13].

Association between thrombolytic treatment and neuropsychological outcomes, particularly VN in the early phases of stroke, has so far received only limited attention. Recent studies suggest that thrombolytic treatment is a significant predictor of earlier discharge to home in patients with moderate/severe RH infarct [14], and it is related to a favourable effect on visuoperceptual functions [15]. Nys et al. [16] found evidence of favourable effect on functional outcome but no effects on any cognitive domain during the 6–10 month followup. They suspected that thrombolytic treatment has a short-term influence on cognitive outcome, but this effect is not sustained or it disappears in long-term followups.

The association between thrombolytic treatment and cognitive functions, particularly VN following RH brain infarct, has not been previously studied. The aims of this study were to explore the presence of VN after RH stroke in the patients with (T+) or without (T−) thrombolytic treatment and to determine whether thrombolysis is a predictor of VN.

## 2. Patients and Methods

### 2.1. Patients

**Figure 1:**
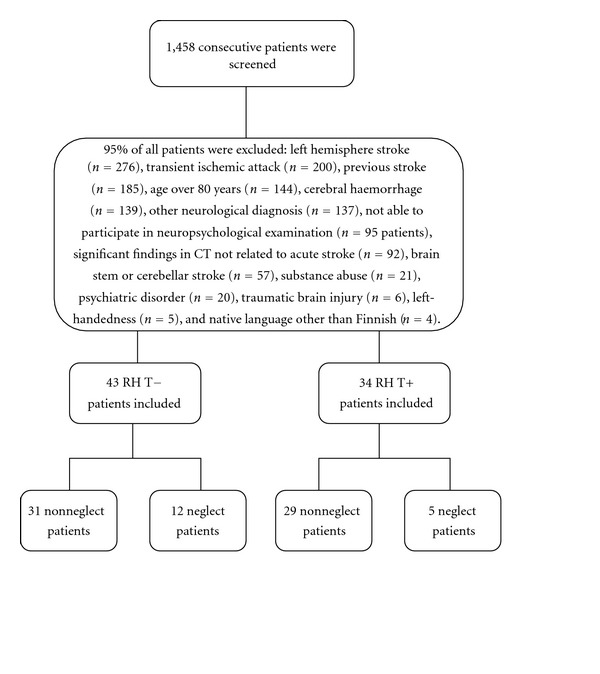
Selection of acute ischemic right hemisphere (RH) stroke patients with (T+) or without (T−) thrombolytic treatment.

We screened 1,458 consecutive patients who were admitted to a university hospital as emergency cases between June 2005 and June 2008. Patients were eligible for inclusion if they had a first-ever ischemic RH stroke. Exclusion criteria were previous history of neurological, cognitive or psychiatric disorders, alcohol abuse, severe primary visual or auditory impairment, left-handedness, decreased level of consciousness, preexistent dependence on activities of daily living, and age over 80 years.  Figure 1 shows the number of patients who were included in this study and the number of who were excluded.

The study was approved by the Ethical Committee of the University Hospital. During their hospital stay all patients received standard treatment. Informed consent was obtained from all participating patients.

### 2.2. Methods

#### 2.2.1. Neuropsychological Examination

The neuropsychological examination was conducted on each patient on average four days (range: 1–11 days) after onset. The general cognitive function was evaluated with the Mini Mental State Examination (MMSE) [17]. Presence of VN was evaluated with the six conventional paper-and-pencil subtests of the Behavioural Inattention Test (BITC) [18]. BITC includes three target cancellation tasks, figure and shape copying, line bisection, and representational drawing. Maximum total score is 146. Patients scoring at or below the cut-off point (≤129) for total BITC score or below the cut-off score on at least two of the six BITC subtests were considered to have VN. For each subtest, we used the same cut-off points as Halligan et al. [19].

#### 2.2.2. Stroke Severity and Computerized Tomography

To define stroke severity, degree of motor defects, and presence of hemianopia, we used the National Institute of Health Stroke Scale (NIHSS) [20]. NIHSS was scored before treatment on arrival at the emergency department (NIHSS at baseline), and later on the neurological ward on average four days (range: 1–10) after onset (NIHSS at neurological ward). Hemiparesis was scored using a scale from 0 (= normal) to 4 (= severe hemiparesis) for leg and arm separately, and these scores were then summed. Hemianopia was assessed using standardized neurological confrontation technique and was scored as absent (0) or present (1).

At the acute stage of stroke, a computerized tomography (CT) of the brain was performed to detect the site of the lesion. Within the first three hours of stroke, thrombolytic treatment was administered as recommended in the National Institute of Neurological Disorders and Stroke study [21].

### 2.3. Statistical Analyses

Since some of the parameters were not normally distributed and the sample sizes were small, we chose to use nonparametric tests for continuous variables. Differences between the T+ and the T− groups in continuous variables were analyzed using the Mann-Whitney *U* test. Categorical variables were compared using crosstabulations.

Logistic regression analysis was used to adjust the association between thrombolytic treatment and VN for other possible factors. In this analysis, thrombolytic treatment, NIHSS at baseline, age, years of education, and gender were used as independent variables and VN as the dependent variable. The results are presented as odds ratios (OR) with corresponding 95% confidence intervals (95% CI). Statistical significance was set at 0.05 for all analyses. All reported *P* values are based on two-tailed tests.

## 3. Results

After application of these exclusion criteria, the final study group consisted of 77 right-handed RH brain infarct patients, 34 received thrombolytic treatment. Patients were divided into the two groups (T− versus T+), and groups did not differ statistically significantly in age, gender, years of education, MMSE, days from onset to neuropsychological or neurological examinations, and presence of hemianopia or hemiparesis. The details of clinical characteristics are shown in Table 1.

### 3.1. The Presence of Visual Neglect

In the neuropsychological examination, 22% of all RH stroke patients had VN. VN was present in 15% of the patients in the T+ group and in 28% of the patients in the T− group, but the difference was not statistically significant (*P* = 0.168). Logistic regression analysis showed that thrombolytic treatment independently predicted absence of VN after adjusting for years of education, gender, age, and NIHSS at baseline. RH patients without thrombolytic treatment had a higher probability of VN than those with thrombolytic treatment (OR = 4.366; 95% CI, 0.994 to 19.175: *P* = 0.05) after adjusting for other factors.

### 3.2. Stroke Severity

On admission to the emergency department, the T+ group showed more severe stroke in NIHSS than the T− group, but this difference was not statistically significant (*P* = 0.137). A statistically significant difference was found between the T− and the T+ groups in NIHSS (*P* = 0.009) on average four days after onset, indicating that patients in the T− group had more severe stroke than patients in the T+ group.

## 4. Discussion

This study was conducted among a homogenous group of consecutive RH ischemic stroke patients. The main aims of this study were to explore the presence of VN after RH stroke in the patients with (T+) or without (T−) thrombolytic treatment, and to determine whether thrombolysis is a predictor of VN.

According to the neuropsychological examination, VN was present in 15% of the patients in the T+ group and in 28% in the T− group, but this difference did not reach statistical significance. Thrombolytic treatment independently predicted absence of VN, and therefore patients in the T− group had a higher probability of VN. NIHSS baseline scores did not differ significantly between the T+ and the T− groups, but after average four days from onset the T− group had statistically significantly higher NIHSS values than the T+ group.

There are no earlier reports on the presence of VN after thrombolytic treatment at the acute phase of stroke. Nys et al. [16] did not find an effect of thrombolytic treatment on any cognitive domain after a minimum of a six-month followup, and they concluded that thrombolytic treatment is associated only with basic functional outcome in the followup. Furthermore, they speculated that thrombolytic treatment might have a short-term influence on cognitive function, but this effect is not sustained at later followup. In our study, we found that thrombolytic treatment had a favourable effect on VN in the very early stages of stroke. Patients in the T− group had a higher risk of VN after average four days from onset than patients in the T+ group.

We found that the presence of VN in the T+ group after RH stroke was lower than indicated in previous studies. According to Bowen et al. [1], the median reported prevalence of neglect in RH patients is 43%. In our study, only 15% of the T+ patients had VN, whereas Jehkonen et al. [2] reported that the presence of VN in the acute phase was 38%. The exclusion criteria, research setting, and the methods used to assess VN in the study of Jehkonen et al. [2] are identical to those in the present study, but at the time of that earlier study thrombolytic treatment was not available at our hospital.

VN was present in 28% of acute RH stroke patients without thrombolytic treatment. Earlier studies on the presence of VN without thrombolytic treatment have reported figures that vary widely from 13% to 82%: this is explained by differences in patient selection criteria, time elapsed since stroke, and the methods used in assessing VN [1]. The differences in the sensitivity of neglect tests [19, 22] and the reported presence of neglect depend on the methods used [23, 24]. Our study was conducted among a homogenous group of consecutive RH stroke patients, and the time elapsed since stroke until assessment of VN did not differ statistically between the T+ and the T− groups. The presence of VN was assessed in each patient on average four days after onset using six standardised subtests of the BITC. In the study by Nys et al. [16], the assessment of VN was based on a single task, and they did not report the presence of neglect in the acute phase of stroke. In the study by Di Legge et al. [25], neglect was detected with NIHSS, which includes only one item to evaluate neglect. It has earlier been confirmed [19, 23] that one test is not enough to determine the presence of neglect. A comprehensive assessment of VN must include different types of measures. The evaluation of VN in the acute phase of stroke before and after thrombolytic treatment must rely on standardized methods, and it is also necessary to followup the patients.

According to Pedersen et al. [4], the presence of neglect is associated with the severity of stroke. Our results here were similar: after four days from onset, NIHSS scores were lower in the T+ group than in the T− group, and similarly VN was more common in the T− group than in the T+ group (28% versus 15%). Baseline NIHSS on admission did not differ statistically significantly between the two groups, but their sum scores for stroke severity were different (median NIHSS score: 6 versus 4). NIHSS scores improved more between admission and four days after onset in the T+ group than in the T− group (median NIHSS score: 6 to 1 versus 4 to 3).

The main strength of this study is that it was carried out in a homogeneous group of consecutive RH patients who had suffered their first brain infarct. Secondly, the presence of VN was assessed in each patient using a systematic battery of standard paper-and-pencil tasks [18] which is in widespread clinical use and focused on assessing extrapersonal neglect in near space. One limitation is the small number of stroke patients in the subgroups. Furthermore, we only investigated RH stroke patients, which means that the results cannot be generalized to the whole stroke population.

Despite these limitations, our results indicate that the administration of thrombolytic treatment within the first three hours of RH stroke decreases the risk of VN. The presence of VN after RH stroke without thrombolytic treatment was 28% and with thrombolytic treatment 15%. We conclude that VN occurs more often in RH infarct patients who do not receive thrombolytic treatment than in those who do receive thrombolytic treatment within the first three hours of first-ever brain infarct, but further research is needed to confirm this result.

## Figures and Tables

**Table 1:** Clinical characteristics of patients with (T+) and without thrombolysis (T−) and the comparison between the groups.

	T+ (*n* = 34)	T– (*n* = 43)	*P* value
Male/Female	19/15	31/12	0.090
Age: Md (range)	60.5 (30–77)	62.0 (36–79)	0.252
Education in years: Md (range)	10.0 (6–16)	9.0 (6–20)	0.278
MMSE: Md (range)	27.5 (20–30)	27 (21–30)^a^	0.731
Neuropsychological examination (days): Md (range)	5.0 (1–10)	3.0 (1–11)	0.798
Neurological examination (days): Md (range)	5.0 (1–10)^b^	3.0 (1–10)^a^	0.305
(a) Baseline measures			
NIHSS: Md (range)	6.0 (1–17)	4.0 (1–15)	0.137
Hemianopia: present (%)	5 (16)^a^	6 (14)	0.841
Hemiparesis: present (%)	26 (76)	30 (70)	0.515
(b) Measures at ward			
BITC: Md (range)	143.5 (38–146)	142 (31–146)	0.150
BITC: VN present (%)	5 (15)	12 (28)	0.168
NIHSS: Md (range)	1 (0–17)^a^	3 (0–14)^b^	0.009
Hemianopia: present (%)	5 (15)^b^	5 (12)^c^	0.745
Hemiparesis: present (%)	11 (33)^b^	17 (40)^c^	0.426

Abbreviations: Md: median; BITC: sum score of six conventional subtests of the Behavioural Inattention Test (range 0–146; ≤129 = visual neglect, ≥130 = no visual neglect); MMSE: Mini Mental State Examination (range 0–30); Neuropsychological examination: days from onset to neuropsychological examination; neurological examination: days from onset to neurological examination; VN: visual neglect; NIHSS: sum score of the National Institute of Health Stroke Scale (range: 0–34; 0 = no defect; 34 = severe stroke); at baseline: outcome on admission in the emergency department; at ward: outcome at neurological ward; ^a^missing value for one patient; ^b^three patients had missing values; ^c^four patients had missing values.
